# Stroke thrombolysis or not for an intraventricular thrombus

**DOI:** 10.1186/s42466-023-00235-x

**Published:** 2023-03-20

**Authors:** Josef Finsterer, Sounira Mehri

**Affiliations:** 1 Neurology and Neurophysiology Center, Postfach 20, 1180 Vienna, Austria; 2 Biochemistry Laboratory, LR12ES05 “Nutrition-Functional Foods and Vascular Health”, Faculty of Medicine, Monastir, Tunisia

**Keywords:** Thrombolysis, Stroke, thrombus, Cardioembolism, Outcome

Letter to the editor

We read with interest the article by Kitmeridou et al. on a 35 years-old male in whom work-up for acute heart failure revealed a thrombus in the left ventricular cavity and who consecutively suffered an acute stroke with aphasia, dysarthria, and right hemisyndrome 24 h after admission [1]. Although computed tomography (CCT) showed no acute lesion but only two old ischemic lesions, systemic thrombolysis was carried out despite the intracardiac thrombus [1]. Magnetic resonance imaging (MRI) 24 h after the acute event revealed acute ischemic embolic lesions in the territories of the left anterior middle cerebral artery (MCA) and both posterior cerebral arteries (PCAs) [1]. The short-term outcome was favourable [1]. The study is excellent but has limitations that raise concerns and should be discussed.

A limitation of the study is that several data regarding the individual and family history were not provided. We should know whether the individual history was positive for stroke or cardiac disease and whether the family history was positive for hereditary cardiac disease, in particular hypertrophic or dilative cardiomyopathy or left ventricular hypertrabeculation (LVHT), also known as non-compaction. LVHT can be complicated by intertrabecular thrombi, which can give rise to cardio-embolism. We should also know whether the two old ischemic strokes seen on the initial cerebral CCT have manifested clinically. Was the individual history positive for arterial hypertension, heart failure, cardiac embolism, or arrhythmias? What was the pre-hospital modified Rankin scale (mRS)? What was the current medication the patient regularly took prior to hospitalisation?

Another limitation is that several data regarding the findings obtained during hospitalisation were not provided. We should know whether C-reactive protein, creatine-kinase (CK), CK-MB, troponin, or pro-brain natriuretic peptide (pro-BNP) were elevated and whether these parameters showed dynamic changes over time. What was the cause of intraventricular thrombus formation? To rule out artery-artery embolism, it is mandatory to carry out a CT of the aorta and carotid ultrasound. Was a CT-angiography with contrast medium carried out? Did the patient ever undergo a cardiac MRI?

Another limitation is that the patient did not undergo acute multimodal cerebral MRI when aphasia and the right-sided hemi-syndrome acutely developed. A cerebral MRI was done not earlier than 24 h after onset of acute neurological deficits and documented acute ischemic lesions in the territory of the left MCA and the PCA bilaterally. Thrombolysis was carried without documentation of the stroke on imaging prior to injection of rtPA. Why was no MRI carried out before thrombolysis to assess the relation between stroke core and penumbra. Whether acute ischemic stroke in three territories resulted from untriggered embolization during fragmentation of the intracardiac thrombus or due to other reasons, remained speculative.

Another limitation is that there is no mention of the long-term cerebral and cardiac outcome. There is also no mention of the long-term anticoagulation regimen and no mention of the long-term clinical outcome of the index patient.

We disagree with the notion that the patient significantly improved after thrombolysis. The NIHSS improved only from 6 to 5 and was 4 at discharge and the mRS was 2 on discharge without knowing the pre-morbid mRS.

The decision for or against systemic thrombolysis in a patient with acute stroke and intracardiac thrombus depends on several cerebral and cardiac prerequisites. Cerebral conditions that should influence the decision include the pre-morbid cognitive status, morphological condition of the brain, previous history of stroke or bleeding, pre-stroke mRS, number and severity of cardiovascular risk factors, location of the stroke, and NIHSS. Cardiac conditions that should influence the decision are the thrombus structure (organised (adherent) or flotile (non-adherent)) and the etiology of intraventricular thrombus formation (heart failure, LVHT, atrial fibrillation, myocardial infarction, endocarditis, coagulopathy, exsiccosis, co-medication).

Overall, the interesting study has limitations that put the results and their interpretation into perspective. Clarifying these limitations would strengthen the conclusions and could improve the study. Indications for or against acute systemic thrombolysis in case of acute ischemic stroke in patients with a ventricular thrombus should rely on several influencing determinants. If available and not contraindicated, thrombectomy should be preferred over thrombolysis in patients with acute ischemic stroke and an intracardiac thrombus.

## Data Availability

All data are available from the corresponding author.
